# Investigation of medical intervention with fatal outcome: the impact of post-mortem CT and CT angiography

**DOI:** 10.1007/s11547-015-0574-5

**Published:** 2015-08-19

**Authors:** Axel Heinemann, Hermann Vogel, Martin Heller, Antonios Tzikas, Klaus Püschel

**Affiliations:** Institute for Legal Medicine, University Medical Center Hamburg-Eppendorf, Butenfeld 34, 22529 Hamburg, Germany

**Keywords:** Medical error, Fatal outcome, Forensic radiology, Post-mortem CT, Post-mortem CT angiography

## Abstract

Post-mortem computed tomography (PMCT) has been proven for its appropriateness to become an integral part of routine pre-autoptic forensic investigations either in the field of forensic investigation of fatal medical error or in hospital quality management. The autoptic investigation of unexpected and peri-interventional deaths can be usefully guided by post-mortem imaging which offers significant added value in the documentation of misplacement of medical devices before dissection with the risk of artificial relocation and the detection of iatrogenic air embolism. Post-mortem CT angiography (PMCTA) augments PMCT in the search for sources of hemorrhages and for the documentation of vascular patency and unimpaired perfusion after general and cardiovascular surgery or transvascular catheter-assisted interventions. Limitations of PMCT and PMCTA in medical error cases are method-related or time-dependent including artifacts by early post-mortem tissue change. Thromboembolic complications including pulmonary embolism, the differentiation of ante- and post-mortem coagulation and the detection of myocardial infarction remain areas with compromised diagnostic efficiency as compared to autopsy. Furthermore, extended survival periods after a complication in question impedes visualization of contrast agent extravasation at vascular leakage sites. PMCT and PMCTA contribute substantially for proving a correct interventional approach and guide forensic or clinical autopsy in the reconstruction of adverse medical events with fatal outcome. Post-mortem imaging could also assume a new role as an alternative in a clinicopathological setting if autopsy is not achievable when the probability in the individual case is acceptable to answer specific questions.

## Introduction

During the last 10 years, post-mortem computed tomography (PMCT) has developed to become a tool for routine work in more and more institutes of forensic medicine across Europe and worldwide [1–8]. PMCT complements the internal examination of the body by being implemented in pre-autopsy routines while new techniques are under development for post-mortem application and the new subspecialty of forensic radiology is developing [9, 10]. There are a lot of forensic scopes of application for imaging methods but PMCT attracts the most attention in the forensic scientific community due to its easier accessability and greater significance in skeletal diagnosis in comparison to other methods. The use of imaging for the detection of errors in treatment dates back to 1895, when it was first accepted as evidence in court [11].

There is a rising number of reports about the advantages of post-mortem imaging in cases with fatal outcome after medical intervention [12–18]. Autopsy can be guided by PMCT not only in case of skeletal trauma but also by typical features like fatal complications after medical treatment such as misplaced catheters, guidewires, tubes and drainages, sources of intervention- related hemorrhages and gas embolism.

Hence, PMCT shows the potential to become a valuable tool that adds further information after unsuspected in-hospital deaths even if an autopsy diagnosis is not achievable due to patient/family consent [15, 19], inoculation risks or ethnic doctrines [20–22]. However, post-mortem imaging has not been established yet as an acknowledged tool for quality control in hospitals—which may be due to the fact that unlike to the forensic pathologists the interdisciplinary cooperation between radiologists and clinical pathologists has not been established yet at a significant level in many countries.

Taking into account the experiences of forensic radiologists and forensic pathologists with the application of post-mortem imaging in the assessment of suspected medical malpractice, PMCT has established its role for radiologically accessible morphological findings which are indicative for or against an iatrogenic injury caused [17]. The limited validity for a range of typical causes of death can be enlarged by combination with CT angiography and magnetic resonance imaging [7, 15, 23, 24].

Since some years, post-mortem CT angiography has expanded the range of options in the reconstruction of potential medical error since it facilitated the search for hemorrhage sources [16] as well as the documentation of an impaired or unimpaired perfusion after different sorts of surgery and intervention [7]. Key criteria for the decision to perform post-mortem imaging and for its interpretation can be obtained from the preliminary inspection of the medical history of a deceased patient [13].

## Methods

In the Hamburg Institute of Legal medicine, over a period of 6 years about 2000 PMCT investigations have been performed after in-hospital deaths. The responsibility of the Institute of Legal Medicine for the morgue includes fatalities with unknown manner of death from the city of Hamburg but also all in-hospital deaths from the university medical center Hamburg-Eppendorf. This enables the post-mortem CT examination on request by clinical colleagues in cases of naturally certified deaths as well as in cases in which public prosecution decides to stop further investigations in primarily certified unclear manner of death. Until 2012, a 4-slice MDCT (MX 8000) have been used, thereafter a 16-slice MDCT (Brilliance, Philips). A whole-body CT from top to thigh (slice thickness 1 mm, pitch 1.5, 130 kV, 180–230 mAs) is the standard; additionally, dedicated scans of the cranium, the brain and the thorax with higher resolution (slice thickness 0.8 mm, pitch 1.0, 130 kV, 180–230 mAS) may complement the exam.

200 hospital fatalities have been examined with multiphase post-mortem CT angiography (MPMCTA) after performance of PMCT according to reported standard methods [25]. The interpretation was made by a board- certified radiologist, assisted by a board-certified forensic pathologist.

## Fatal outcome in clinical settings: common case groups

### Intensive care unit and emergency medicine

**Fig. 1:**
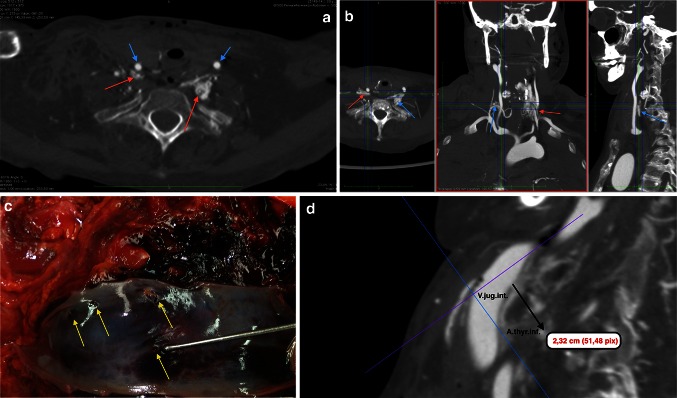
**a**–**d** A 39-year-old female patient received dialysis due to rheumatoid arthritis and secondary amyloidosis and longterm corticoid treatment with terminal renal failure. She was hospitalized for a temporary change to a Sheldon-catheter after a Cimino fistula for dialysis had obliterated. The catheter was placed under ultrasound guidance and heparinisation. After a sudden swelling of the neck and dyspnoea occurred, a tracheotomy was performed because of intricate standard intubation due to rheumatoid cervical spine deformation. Cardiac failure and unsuccessful resuscitation for 30 min. **a** Axial oblique reconstruction, MPMCTA, arterial phase with 500 ml Angiofil^®^; MIP. Contrast medium extravasation surrounding the left and right inferior thyroid artery (*red arrows*). Common carotid arteries (*red arrows*); **b** Orthograde MPR reconstruction demonstrating origin of the injured arteries from the thyrocervical trunk (*red arrow*: *left/blue arrow*: *right* inf. thyr. artery); **c** Four lacerations of the internal jugular vein (autopsy finding); **d** 3D-MPR with oblique reconstruction of the distance between inferior laceration of internal jugular vein and right inferior thyroid artery. *Conclusion* Obviously, the aberrant catheter tip damaged the right, but also left inferior thyroid artery crossing even the midline of the neck (there was no indication for an additional *leftside* skin lesion or jugular puncture)

Post-mortem computed tomography contributes greatly to perpetuate evidence of the position of foreign bodies at time of death and before any autopsy procedure could artificially displace it. The unrecognized misplaced intubation includes esophageal positioning, bronchial intubation, esophageal or tracheal perforation but also disconnection or dislodgement of the tracheal tube. Extra-anatomical gas detection such as gas accumulation in the deep cervical and mediastinal tissues points to a complication, occasionally seen after misplaced tracheotomy or coniotomy. It remains challenging in PMCT to detect a source of gas/air escapes, especially in the thoracic and abdominal cavity but following their 3D-path in soft tissues has advantages over the autopsy. PMCT gives evidence of pneumothorax after jugular and subclavicular medical puncture; the mediastinal shift due to tension pneumothorax cannot be depicted at autopsy in a comparable quality. The transvenous placement of central venous lines, gastric tubes and electrodes from cardiac pacemakers or defibrillators with irregular loops and bending or misplaced pleural drainages are quite illustrative on 3D reconstructions. Figure 1a–d illustrate the misplacement of a Sheldon catheter perforating bilaterally small arterial branches at the base of the neck resulting in massive swelling with fatal upper venous congestion and respiratory failure.

In intensive care units, ventricular assist devices, aortic balloon pumps and extracorporal membrane oxygenation (ECMO) systems have become increasingly in use in patients with cardiac and respiratory failure as a bridge for recovery, transplantation or ultima ratio. PMCT easily identifies the position of the incoming and outgoing components as well as the location of pump systems. Depending on the type of system used, conclusions can be drawn on the underlying organ dysfunction in the terminal phase of life of the patient (left or right ventricular assist, veno-venous for respiratory or veno-arterial ECMO for cardiac support).

### Coronary angiography and transvascular cardiac interventions

After percutaneous coronary intervention (PCI), the myocard sometimes shows an enhancement which seems to be due to contrast medium deposits that still have not been washed out completely. If these areas correspond to the drainage of a coronary artery, this may be taken as an indication for sudden interruption of coronary perfusion before death or immediate cardiac arrest after contrast medium application.

If a series of gas bubbles is observed in a coronary artery after fatal outcome during a PCI procedure, it should be clarified if a gas embolism could have happened even though safety devices should prevent unobserved gas bubble transport through the angiography catheter. Post-mortem gas formation is a very unlikely explanation when gas detection is restricted to the coronary arteries. Another differential diagnosis is the replacement of blood by gas via unlocked catheters which had not been removed from the body immediately after the procedure.

**Fig. 2:**
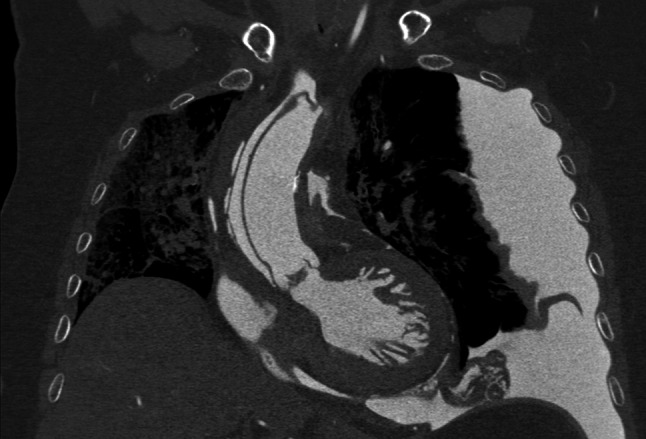
A 71-year-old female patient was admitted in a hospital emergency room due to severe chest pain. On the day after acute cardiac failure occurred during coronary catheterization which had been performed due to abnormalities in ECG. *Diagnosis* aortic dissection type De Bakey I originating in the ascending aorta, propagating in the innominate artery and thoracoabdominal aorta until the bifurcation, perforating into the pericardial sac, mediastinum and left pleural cavity. Thoracic post-mortem CT after MPMCTA (dynamic phase). PMCT, coronal reconstruction, MIP. Autopsy confirmed no relationship to percutaneous coronary intervention

Coronary perforations with cardiac tamponade and dissections are typical fatal complications after transvascular cardiac interventions, sometimes resulting in aortic dissections (Fig. 2). Perforations result from balloon expansions of the arterial wall or stent expansion, at times directly from a catheter tip in a vulnerable vessel wall [13]. Emergency treatment with covered stents in the position of the rupture of the vessel wall may impede the post-mortem demonstration of leakage sometimes; if not, the bleeding, its amount and its origin can be detected.

PMCTA may rarely fail to demonstrate the exact source of a bleeding which resulted in a cardiac tamponade. The tamponade is usually firmly clotted in these cases and prevents contrast medium from leaking out of a rupture due to the massive pressure in the pericardial sac and adherence of blood clots to the surface of the traumatized tissue.

Transvascular valve placement refers predominantly to the transcatheter aortic valve implantation (TAVI) which utilizes bioprostheses made of bovine pericardium mounted on a balloon-expandable stainless-steel or cobalt-chromium stent. The positioning of the valve, its relation to the coronary ostia with potential constriction effects and the unimpeded perfusion of the aortic outlet as well as signs of incompatibility of the aortic diameter and the expanded frame with the risk of valve insufficiency can be visualized by PMCTA. The expanded wires of the frame may complicate a subsequent coronary stenting procedure. The lower border of the valve graft is at risk to interfere with the anterior sail of the mitral valve and its papillary suspension which is observable via 3D reconstruction and virtual endoscopy [13].

The valve may be implanted via a transfemoral or transapical approach which could result into additional suturing problems at the points of sheath/catheter entry after termination of the intervention. PMCT/PMCTA demonstrates resulting hemorrhages into the soft tissues of thigh and retroperitoneum (transfemoral approach) or into the pleural space and into the mediastinum (transapical).

A catheter-induced trauma may also refer to balloon dilatations of the aortic valve, to transcatheter approaches of the mitral valve such as annuloplasty and mitraclip^®^ or to catheter radiofrequency or cryoablations, mostly at the level of the openings of the pulmonary veins into the left atrium for the treatment of cardiac arrhythmias.

These procedures may result into

- Perforations from one into another cardiac chamber.
- Myocardial contrast medium deposits in case of incomplete disruptions.
- Perforation into the pericardial sac with resulting tamponade.
- Perforation into the mediastinum and/or the pleural space if the rupture continues above the level of the pericardial reflection lines on the great vessels.

PMCTA facilitates the diagnosis by following the path of extravasated contrast agent.

### Endovascular aneurysm repair

Endovascular aneurysm repair (EVAR) has been widely adopted for abdominal aneurysms—extendable into the iliac arteries—and thoracic (TEVAR: thoracic EVAR) aortic aneurysms. The blood flows through the endograft which represents an artificial lumen reducing the pressure in the aneurysm. There is a growing application of endovascular graft stenting even in emergencies with ongoing aneurysm ruptures or aortic dissections.

**Fig. 3:**
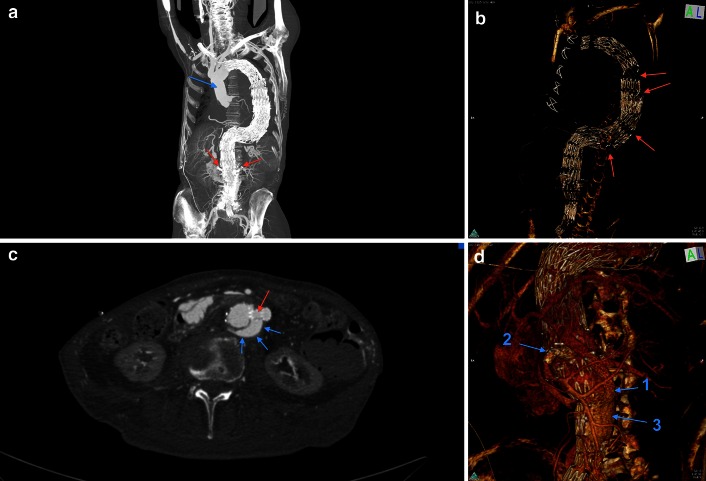
**a**–**d** A 76-year-old male patient had received a hybrid intervention due to an aneurysm of the thoracic and abdominal aorta 2 years before he died from cardiac and respiratory failure. **a** Fenestrated EVAR with 8 sequential endografts ranging from the left subclavian artery down to extensions for the common iliac arteries. Arterial phase of MPMCTA, *front view*, MIP. *Blue arrow* replacement of ascending aorta, major thoracic branches sewed on the prosthesis; *red arrows* fenestrations for renal arteries, reinforced with proximal stents (snorkel/periscope technique). **b** 3D reconstruction with signs of endograft migrations in the large thoraco-abdominal kinking zone (*red arrows*). **c** Celiac bypass with snorkel stent (*red*), endoleak (*blue arrows*). **d** 3D reconstruction in a front left view with celiac artery bypass (*1*), superior mesenteric artery snorkel (*2*) and extravasation of contrast agent [(*3*), endoleakage]

A major cause of complications in abdominal EVAR is the failure of the seal between the proximal, infra-renal aneurysm neck and the endovascular graft; unsuitable fit between the aortic wall and the graft with compromised seal and an instable anatomy may elevate the risk [26, 27]. Recent technology introduced enlargements of the grafts including supra-renal abdominal and thoracic portions of the aorta, branched EVAR combined with fenestrations (FEVAR, Fig. 3a–d) to maintain the patency of visceral and thoracic arteries, chimneys, snorkels or direct fixation and sealing (EndoAnchoring). Hybrid interventions allow positioning of endovascular grafts over major aortic branches by combination with open surgical bypass grafts or replacement of the ascending aorta (Fig. 3a). Many periprocedural deaths are due to acute heart failure following elevated intra-aortic compressive stress or secondary dissections which are facilitated by kinking of the iliac arteries or excessive angulation of the aorta (Fig. 3a, b). PMCTA allows the analysis of the patency of indispensable aortic branches or surgical bypasses and visualizes so-called endoleaks (Fig. 3c, d), which result in a reperfusion of aneurysms or dislocation of the prosthesis. In general, PMCTA demonstrates a high cardiovascular morbidity including cerebral infarctions in these patients.

### General and cardiothoracic surgery

The diagnostic value of PMCT(A) after fatal outcome of open surgical interventions is generally much higher with the detailed knowledge of the preexisting morbidity, the sequence of ante-mortem interventions and preceding complications to the last operation. Full access to the patient history at the time of radiology report would be a given in clinical pathology, but it is not self-evident in the forensic area for the time of post-mortem reporting.

**Fig. 4:**
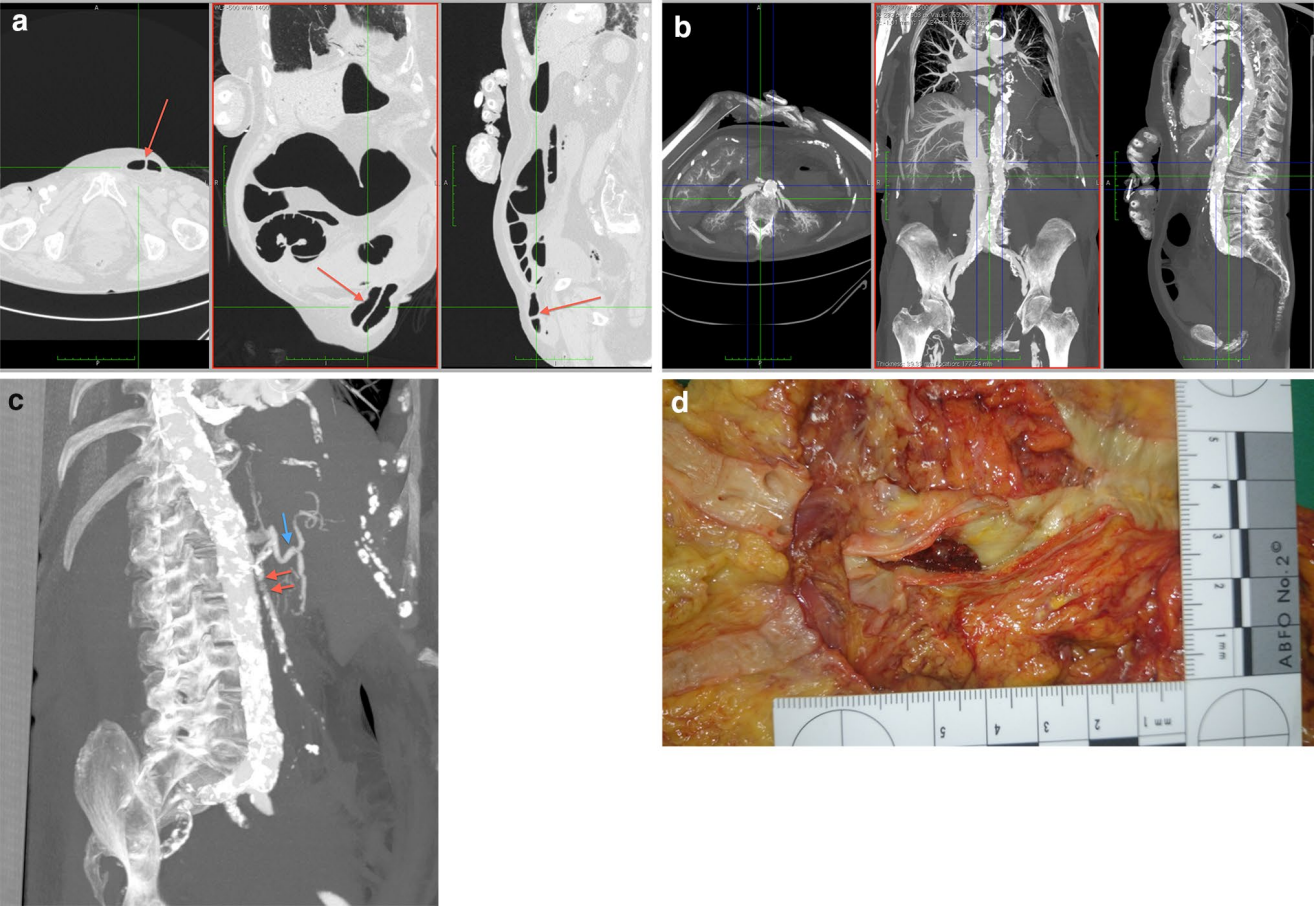
**a**–**d** A 94-year-old female patient was admitted to hospital with epigastric pain and ground vomiting the day before. *Diagnosis on admission* leftside inguinal hernia, no convincing indication for ileus/acute abdomen. While the consiliary surgeon decided to wait and see, laboratory markers indicated myocardial ischemia. On the second day after admission, a transesophageal echocardiography was performed with propofol sedation. 2 h later the patient was found dead on the ward with apparent preceding fecal vomiting. **a** Dilated intestine with inguinal hernia (*red arrow*) native PMCT, MPR; **b** MPMCTA, dynamic phase, MIP. Severe overall calcification of the aorta. Inferior vena cava and liver venes. Note the missing contrast enhancement of the mesenteric vasculature; **c** MPMCTA, arterial phase, MIP. Aorta with celiac artery and common hepatic artery (*blue arrow*). Superior mesenteric artery with severe sclerosis, proximal faint opacification (*red arrows*), ending without visualization of major branches. Inferior mesenteric artery missing either. **d** Fresh thrombosis of proximal mesenteric artery (autopsy finding). The patient died of paralytic ileus due to mesenteric infarction which had been accompanied by cardiac failure with myocardial ischemia

A typical scenario of suspected medical error is a challenged decision to wait and see instead of starting surgical exploration in case of abdominal pain. Figure 4a–d illustrates a case with unexpected death after inconclusive diagnostic approaches.

The majority of patients following thoracotomy or laparotomy-based adverse events die of protracted multi-organ failure with circulatory shock. PMCT shows laparotomy-related complications of abdominal surgery such as postoperative ischemia of the bowel with indirect radiological signs as in the living, a paralytic ileus with bowel distension and the presence of multiple gas–fluid levels. Peritonitis from suture insufficiency after bowel resection, postoperative respiratory failure with pneumonia, wound infections with abscess formation or pyelonephritis are suspected reasons for a septic multi-organ dysfunction syndrome but mostly PMCT offers indirect signs of these conditions only.

General soft tissue edema as well as fluid in the peritoneal and pleural cavities is indicative of failure of kidneys, heart and liver. Maximum pulmonary edema without major post-mortem change and pleural effusions demonstrate respiratory failure and sometimes overhydration. The extracardial distribution of contrast medium deposits in the kidneys or in extra-renal organs indicate ante-mortem contrast enhanced imaging in excretion mode-dependent short-term intervals before death and creates an impression of the extent of renal failure.

Hemorrhages from dehiscent vessel anastomoses or ligatures after resections of abdominal organs are visualized; however, a collapse of major vessels as a sign of hemorrhagic shock is usually missing because of massive transfusion or hyperhydration.

Liver failure is associated in PMCTA with a general elevated tissue enhancement and simultaneous enhancement of arterial and portal structures on the arterial phase images indicating the presence of open arterioportal venous shunts.

**Fig. 5:**
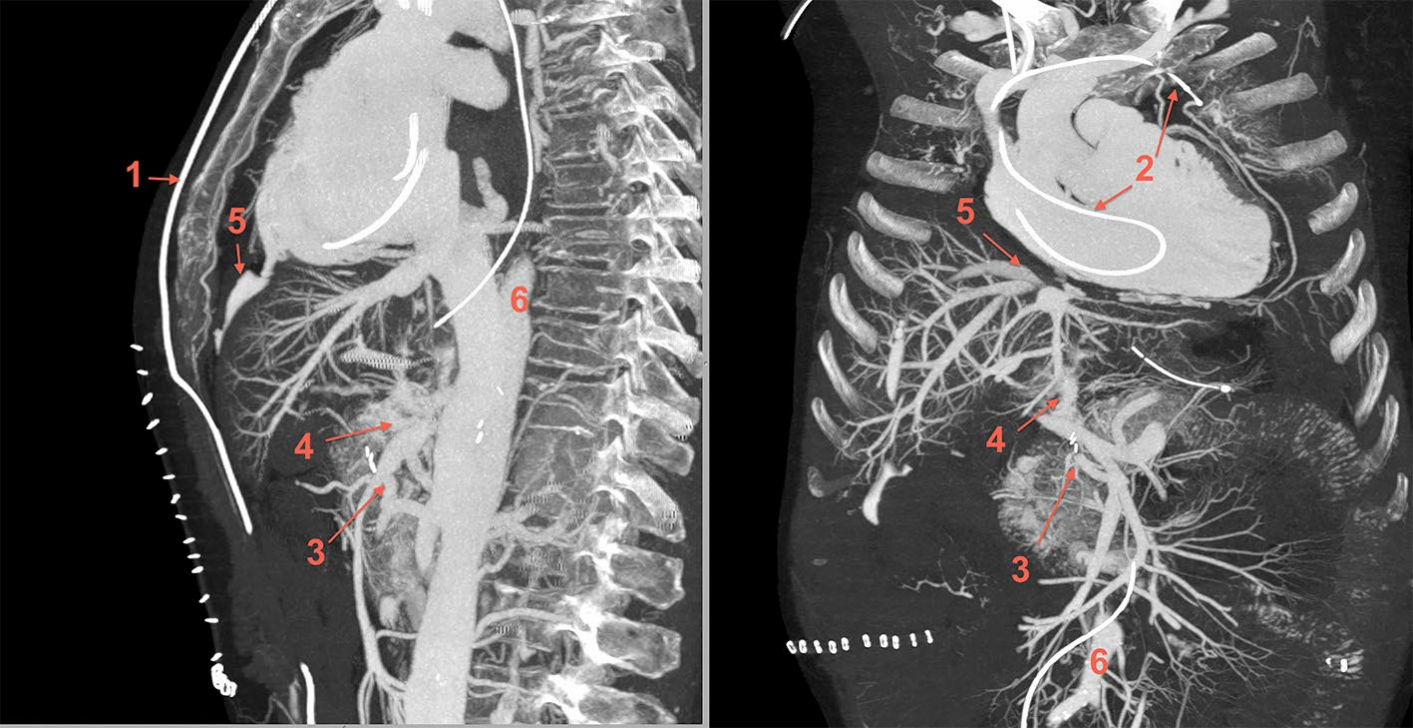
A 72-year-old male patient, liver transplantation, intraoperative circulatory failure due to reperfusion syndrome and transplant failure. MPMCTA, dynamic phase, MIP in sagittal and coronal planes. Ventriculo-peritoneal shunt for decompression of hydrocephalus after subarachnoid hemorrhage (*1*). Displaced catheter for haemodynamic monitoring in the right atrium with irregular loops (*2*), the tip finally propagating retrogradely through the superior vena cava and the brachiocephalic vein into the left superior mediastinum. Disconnected hepatic artery anastomosis with ligated recipient’s supplying artery (*3*) as an ultima ratio for intraoperative temporary limitation of reperfusion effects (in the intraoperative state, the donor’s proximal common hepatic artery had been sewed on a branch of the recipients’ superior mesenteric artery). Note the exclusive portal and hepatic vein opacification of the liver. Leakage of the portal vein anastomosis (*4*) and hepatic vein anastomosis with local subdiaphragmatic hemorrhage (*5*). Aorta (*6*)

Fatal outcome after preceding liver transplantation could be due to hepatic artery/portal vein stenosis or thrombosis which are caused by rejection, clamp injury, intimal injury by perfusion catheters or anastomotic ischemia due to a disrupted vasa vasorum. Figure 5 demonstrates a dehiscence with hemorrhage at the site of the portal vein anastomosis and a disconnected hepatic artery anastomosis in a patient dying in the operating room of acute transplant failure with reperfusion syndrome.

Coronary bypass surgery includes aortocoronary venous bypasses, left internal mammary artery (LIMA) and right internal mammary artery (RIMA) bypasses which are identified in PMCT by clips in characteristic formation [12]. PMCTA illustrates the patency of the vessel as well as of calcifications and stents in the graft.

### Gastroenterology

The diagnosis of gastric or duodenal ulcers could involve the forensic question how successful a laceration of a bleeding artery at the ulcer bed has been terminated by gastroduodenoscopy. Even at autopsy given the sutured ulcer bed this question may be difficult to assess if gastric and bowel contents do not allow to differentiate within a short time interval. PMCTA helps to identify the sufficiency of vessel closure if not hindered by unspecific contrast extravasation due to post-mortem gastric mucosal destruction.

Endoscopic retrograde cholangiopancreatography (ERCP) may be complicated by a rupture of bile duct or pancreatic duct which causes post-ERCP pancreatitis, often promoted by papillotomy or by sampling of suspicious adjacent tissues by endosonographically guided fine-needle aspiration. The post-mortem PMCT diagnostic criteria are direct and indirect signs of bleeding, edema of pancreas and surrounding tissues and accentuation of the renal fascia; in exceptional cases this is combined with the risk of air embolism if gas insufflation into the duct system has not been stopped in time.

Specific complications such as a compromised organ perfusion by relocation of vessel disposals should be proved by means of MPMCTA. This is also true for hemodynamically significant dissection or leakage.

### Neurosurgery

**Fig. 6:**
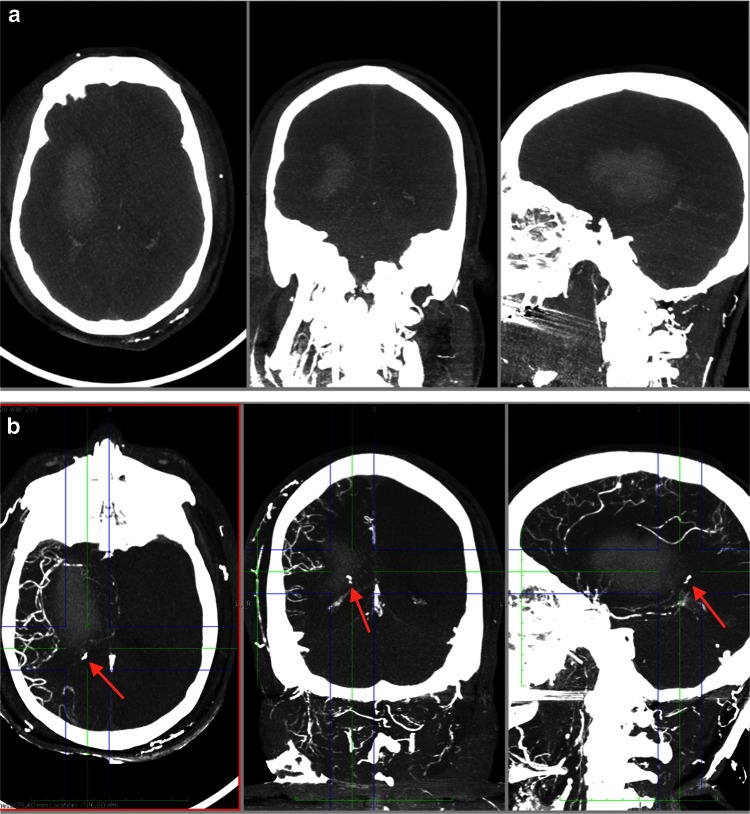
Male, 52 years. The patient suffered from arterial hypertension. Fatal outcome in hospital due to intracerebral hemorrhage after neurosurgical insertion of a probe for intracranial pressure measurement. **a** MPMCTA, standard protocol with femoral arterial and venous access: dynamic phase, MIP. Right hemispheric intracerebral hemorrhage with propagation into the ventricle. **b** MIP after cannulation of right common carotid artery and additional application Angiofil^®^. Note the contrast agent enhancement in the right posterior lobe (*arrows*) representing a potential source of hemorrhage

PMCT after neurosurgical interventions refers to hemorrhage, stroke and traumatic brain injury, the latter indicated by fracture lines, coup and contrecoup lesions. The early post-mortem change with dedifferentiaton of gray and white matter of the brain in PMCT as well as an impaired rendering in the posterior cranial fossa limits sometimes the diagnostic validity and post-mortem MRI could sometimes be the better option for guidance of neuropathological examinations. The enhancement of the intracranial vessels with the MPMCTA transfemoral approach can be blocked by an increased brain pressure. A pronounced block may serve as an indication for intravital brain death. It is possible in some cases to overcome this effect by direct cannulation of the great cervical vessels for contrast agent application (Fig. 6a, b).

## Discussion

Medical malpractice allegations after deaths were between 1990 and 2000 in Germany occasion for approximately 4.4 % of the judicial autopsies; the trend is likely to be increasing since then [28]. The usual procedure in the majority of cases is a forensic autopsy the result of which is commonly used with the preliminary information from the medical files of the patient to decide whether a peer review for the final evaluation of the case in question is needed.

If the patient dies from the effects of treatment failure, negligent homicide, grievous bodily harm with lethal consequences or even manslaughter come into consideration. Among the typical error categories are found primarily the omission of necessary diagnostic or therapeutic procedures and perioperative complications [28]. The forensic clarification of medical malpractice allegations frequently ends up in exculpatory expertises. Nevertheless, this process has an acknowledged effect with regard to secondary medical error prevention [29].

Frequent underlying causes of medical errors are lack of “error culture”, possibilities for confusion (drugs, right–left confusion, mistake of patients), communication errors among those administering treatment, workload, lack of clarity about the responsibilities and inadequate patient monitoring. Typical case scenarios of unexpected death in hospital, sometimes in the context of medical malpractice allegations, are sudden and fatal deteriorations after surgical and minimally invasive intervention, delayed or fault diagnosis and treatment, death after failed resuscitation (suspicion of delayed action, inadequate performance, particularly problems with intubation), and generally a mismatch between low estimated and risk fatal outcome. All of these are typical scenarios for a potential added value of a multimodal post-mortem analysis including imaging methods. However, under-/overdosage of medical drugs necessitate toxicological investigations and allergic reactions to medical drugs and contrast agents will be evaluated mainly by medical history and laboratory markers.

A decline of clinical autopsies results in an overestimation of the sensitivity of clinical ante-mortem diagnosis [21]. Clinicopathological studies with MRI have been reported [30], some with special respect to pediatric pathology [31, 32]. The application of PMCT in case of deceased patients from an Intensive Care Unit at a University Medical Center shows a high accordance rate of clinical “major” and “minor” diagnoses between imaging and autopsy results and PMCT may supplement clinical diagnoses in cases of missing autopsy [17]. The same setting was investigated for the efficiency of PMCTA—this study found a confirmation rate of 93 % of all diagnoses identified from ante-mortem medical records for PMCT/PMCTA as compared to a rate of 80 % for conventional autopsy. The higher accordance remained true even in the subcategory of cardiovascular diagnoses; however, a major limitation was still that ante-mortem known myocardial infarctions were confirmed in PMCTA by potential coronary culprit lesions only [18].

MPMCTA turns out to be a new method with a highly standardized and evaluated methodology [7, 25, 33] which is more easily implementable in pre-autopsy practice than post-mortem cast angiography with silicone which also proved to discover graft twists after bypass surgery remaining undetected at autopsy [34], to detect fresh cerebral infarctions as well as neurovascular main stem thrombosis, aneurysms and arteriovenous malformations [14, 34, 35, 36]. In case of infectious complications imaging can facilitate sterile post-mortem puncture of radiologically suspicious areas for microbiological investigations before autopsy [37].

Multiphase post-mortem CT angiography contributes to the detection of hemorrhage sources even in cases in which autopsy fails [7, 14]. Blood coagulation disorders are a frequent condition in the case group of hemorrhage complications under suspicion of medical error. Hence, as compared to trauma cases in forensic daily routine medical error cases often challenge the forensic pathologist at autopsy with the preponderance of

- discrete bleeding sources in small arterial branches
- venous leakages without recognizable lacerations of small venes/venoles, which is typical for the renal bed, mesenterial veins and venous plexus in the pelvic region
- bleedings through miniscule defects caused by perforations with tiny instruments such as catheter tips
- multiple bleeding sources which succeed each other at various time points

The concept of MPMCTA distinguishes arterial and venous sources nevertheless early venous enhancement does occur in the arterial phase, particularly in the portal vein [38]. The intensity of the leakage of contrast medium into body cavities frequently corresponds to the extent of vessel damage; however, this is not necessarily the case in soft tissues when ante-mortem extravascular hemorrhage exerts a compressive effect or has coagulated.

Due to the reluctance of medical doctors concerning informed autopsy consent and inadequate prioritization by the legislature [39] post-mortem imaging should assume a new role as an alternative. Furthermore, it could serve as a screening tool guiding the discussion on how to decide about a more detailed forensic or clinicopathological examination [1]. This could apply for unexpected deaths in the context of medical treatment; however, the necessary standardization of protocols for PMCT and post-mortem magnetic resonance imaging (MRI) is still pending [21].

### Limitations of PMCT

The evidence for an isolated use of the results of post-mortem imaging in forensic cases is still limited: autopsy remains the gold standard for the morphological diagnosis of thromboembolic complications as post-mortem imaging is restricted to probabilistic approaches concerning the quality of obliterating material in a vessel. Myocardial infarction remains an autopsy- and histology-based diagnosis [30] even if MPMCTA indicates sometimes myocardial damage and post-mortem MRI could help with promising indications for peracute myocardial damage [40].

Another obstacle is the early post-mortem change [41] with hypostatic fluid transgression between different anatomic compartments which result in diagnostic pitfalls in the radiological assessment of the lung. Depicting the heart by PMCT at different post-mortem intervals demonstrates considerable changes in volume and tension as well as alterations in shape and position either; for the diagnosis of terminal or chronic dilation, dilatative and other cardiomyopathies a degree of caution is strongly advisable [13].

Arrhythmogenic cardiac deaths due to coronary heart disease are a further example of the limits of current post-mortem imaging [17]. Autopsy also fails in this regard if the histological examination of the conduction system remains inconclusive. However, the conventional approach to certify a “sudden cardiac death” by safeguarded exclusion of a common standard set of alternative causes of death would be unreliable based solely on imaging.

## Conclusions

Post-mortem cross-sectional imaging has demonstrated essential benefits as an adjunct to forensic autopsy. Enhanced by angiography methods, it facilitates the reconstruction of anatomical conditions in the region of an adverse event during operation. This refers to the anatomical proximity of unintentionally injured structures, the search for sources of bleeding as well as the question of perfusion delivery after vascular procedures. The availability of these techniques should be considered in appropriate cases.

Post-mortem imaging allows comparisons with ante-mortem imaging in individual cases and could help in the expert’s delimitation of ex ante and ex-post judgements by clarification of situational conditions in the very moment of a challenged medical action or decision.

Taking into account the still limited availability of expertise in post-mortem radiology, a cooperative assessment via teleradiology expert networks could be a preferable option [42].

The combination of imaging and autopsy, histology and toxicology could enhance the cost efficiency of criminal proceedings by accelerated setting the course and avoidance of costly follow-up assessments [14]. Furthermore, multimodal post-mortem examinations provide—beyond the forensic scope—a new opportunity to place an emphasis on clinical mortality analysis according to quality management principles.
